# Retaining a Multicultural Nursing Workforce: A Self-Determination Theory Perspective

**DOI:** 10.1177/10436596251318027

**Published:** 2025-02-14

**Authors:** Princess Villamin, Violeta Lopez, Deependra Kaji Thapa, Michelle Cleary

**Affiliations:** 1 CQUniversity, Australia; 2 Indiana University Bloomington, USA

**Keywords:** nurse migration, nurse retention, workforce diversity, organizational culture, management, self-determination theory

## Abstract

**Introduction:**

The increased globalization of nurses has prompted organizations to explore innovative strategies to retain their workforce. However, due to cultural variations and increased workforce diversity, a one-size-fits-all retention strategy may not be effective.

**Methodology:**

In this paper, we discuss nurse migration and retention to identify points of intersection and possible theories that can be applied.

**Results:**

Nurse migration and retention share a common motivation thread, indicating that a motivation theory could effectively integrate both concepts. Self-determination theory (SDT) is particularly relevant as it suggests that the needs for autonomy, competence, and relatedness influence motivation and retention and that these are universal, transcending cultural boundaries.

**Discussion:**

Addressing migrant nurse retention is crucial. The continued international recruitment poses a threat, as any intake by host countries further depletes the already scarce pool of nurses in some source countries. The perspective offered by the SDT may prove instrumental in developing effective strategies for retaining migrant nurses.

## Introduction

The increasing mobility and migration of nurses pose a challenge in the current climate of nursing workforce shortages (Buchan & Catton, 2023; Shaffer et al., 2022; Villamin et al., 2024). Previous literature has used micro and macro perspectives to understand retention and turnover among nurses (Gilmartin, 2013; Hayes et al., 2012; Kim & Kim, 2021; Nei et al., 2015; Woodward & Willgerodt, 2022). However, it is essential to consider theories on migration and retention that examine the economic, professional, social, political, and personal factors (Brewer & Kovner, 2013; Dywili et al., 2013; Toyin-Thomas et al., 2023) that contribute to a nurse’s decision to migrate and stay post-migration in a host country. It is also important to consider the cultural relevance of theories, where any studies on migrant nurses may comprise nurses from collectivist and individualist cultures. This article discusses the value of using the self-determination theory (SDT) within a qualitative paradigm for this purpose. This paper provides an overview of theories used in nurse migration and retention studies, followed by a discussion of how the principles of SDT bridge the gap between concepts of migration and retention, and how this theory applies to individualist and collectivist cultures. Finally, a conceptual framework, including the SDT within a qualitative paradigm, is presented to better understand retention among migrant nurses.

## Background

The global workforce deficit, primarily driven by shortages in nurses and midwives who make up more than 50% of the total shortfall, poses a significant threat to global health (International Council of Nurses [ICN], 2022; World Health Organization [WHO], 2022). Globally, the demand for nurses is expected to rise due to various factors, such as an aging workforce, workforce pressures from the chronic nursing shortage, longer life expectancies, and an increase in chronic health conditions (Health Workforce Australia, 2014; Kovner, 2022; Mannix, 2021). The ICN urges countries to be self-reliant and increase their supply of domestic nurses; however, international recruitment is the “quick fix” for workforce shortages, especially among high-income countries (Buchan & Catton, 2023).

The international mobility of nurses has highlighted retention issues among source and host countries (Pressley et al., 2022). For instance, in New Zealand, migrant Asian nurses were found to consider onward migration to another host country or returning to their respective source countries between 2 and 5 years after their migration to New Zealand (Walker & Clendon, 2015). Similar findings are reported in the United Kingdom, where 23,434 migrant nurses and midwives deregistered between 2014 and 2019, with the most frequent reason being to leave the country (Nursing & Midwifery Council, 2019). Host countries may continuously recruit internationally to fill their workforce shortages (ICN, 2021; Shaffer et al., 2022; Villamin et al., 2024), where any intake poses the risk of recruitment from an already depleted pool of nurses elsewhere. As the flow of international nurses increases (Buchan & Catton, 2023), the issue of retention, including among migrant nurses, becomes even more significant.

## Nurse Migration

Migration can be broadly defined as a temporary or permanent change in residence both within (internal migration) and outside borders (international migration; Brettell & Hollifield, 2023; E. S. Lee, 1966; Massey et al., 1993). Nurse migration involves the movement of nurses from one country to another for various reasons. Defining attributes of nurse migration identified in a concept analysis include the motivation and individual decision to migrate, barriers and facilitators, freedom to migrate, and dynamic movement (Freeman et al., 2012).

The study of migration calls for a multidisciplinary approach to capture the diversity, forms, factors, motivations, and contexts in which migration occurs (Arango, 2000; Brettell & Hollifield, 2023; Massey et al., 1993). Although there are multiple migration theories from numerous disciplines (Brettell & Hollifield, 2023), the most commonly adapted for understanding nurse migration is the push–pull theory (Freeman et al., 2012; Kingma, 2007; Mejia et al., 1979; Toyin-Thomas et al., 2023).

The push–pull theory states that there is a presence of factors that push migrants from source countries (e.g., labor market, political instability) and factors that pull migrants into host countries (e.g., opportunity, better wages, and lifestyle; Palmer et al., 2021; Sands et al., 2020; Toyin-Thomas et al., 2023). In a report for the World Health Organization, Mejia et al. (1979) considered the theory a truism with nurse migration only occurring in the presence of both push and pull factors. The economic and political factors of the push–pull theory may explain the migration of nurses from low-income to middle- and high-income countries, but this may not explain other patterns of nurse migration, including onward migration, return migration, or the migration of nurses between middle- and high-income countries. This theory simplifies the complexity of nurse migration, where studies need to integrate the role of social and cultural differences that underlie patterns and influence nurses’ migration experiences (Prescott & Nichter, 2014).

The increasing globalization of nurses presents two views on nurse migration: the initial migration of nurses from their source countries and the onward migration of migrant nurses from host countries. Although these are separate concepts, they also intersect in that the reasons for initial migration may be similar (or not) to reasons for onward migration. Thus, although the push–pull theory has provided insight into why nurses initially migrate from their source countries, it is unclear whether this accounts for why nurses leave their host countries (again). To maintain clarity, this paper will focus on nurses who migrated from their source countries, termed migrant nurses. However, some discussions may apply to both migrant nurses and nurses who are yet to migrate.

## Nurse Retention

To understand the onward migration of nurses, it would be remiss not to include studies on nurse retention. Simply put, the retention of migrant nurses may equate to the absence of onward migration. Nurse retention has four defining attributes: individual motivation and decision, strategy and intervention, geographic context, and work attachment (Efendi et al., 2019). Retention has been studied using various theoretical frameworks, including Maslow’s hierarchy of needs (Chiao et al., 2021; Paris & Terhaar, 2010), Herzberg’s motivation-hygiene theory (J. Y. Lee & Lee, 2022; Rai et al., 2021), and social exchange theory (Shacklock et al., 2014; Trybou et al., 2014). Maslow’s hierarchy of needs (Maslow, 1943) suggests that individual needs are arranged in a hierarchy, where the lower-level needs, such as food, water, and shelter, should be met before individuals progress toward higher-level needs, such as security, belonging, and self-actualization. As individuals have an innate motivation for growth and development, they are motivated to achieve unmet needs and remain motivated as they progress toward higher-level needs (Paris & Terhaar, 2010). Herzberg’s motivation-hygiene theory (Herzberg et al., 1959) expanded on Maslow’s hierarchy of needs, suggesting that there is a duality of factors that either lead to job satisfaction (motivators) or dissatisfaction (hygiene factors; Alrawahi et al., 2020; Alshmemri et al., 2017). These two theories have informed studies on nurse retention and reported that when organizations can meet nurses’ needs (Maslow’s hierarchy) or when they can strengthen motivators and decrease hygiene factors, nurses are more likely to remain (Chiao et al., 2021; J. Y. Lee & Lee, 2022; Paris & Terhaar, 2010; Rai et al., 2021).

The social exchange theory provides another perspective by focusing on the relationships individuals have with their organizations (Blau, 1964; Copranzano & Mitchell, 2005), theorizing that individuals who have positive relationships with their supervisors have positive work-related behavior while the opposite occurs with individuals with negative relationships with their supervisors (Shacklock et al., 2014; Trybou et al., 2014). When used to understand nurse retention, this theory posits that positive relationships result in organizational commitment and job satisfaction, which are linked to nurse retention (Shacklock et al., 2014; Trybou et al., 2014).

Although several theories have been used to explore nurse retention, studies applying these theories have not differentiated between migrant and non-migrant nurses. It is, therefore, unclear how relevant these theories are to studying retention among migrant nurses. It cannot be assumed that the reason for migration will be the same reasons for retention when these are met, especially considering migrant nurses’ cultural backgrounds, motivations, and individual circumstances (Villamin et al., 2024).

## Nurse Migration, Retention, and Motivation

Concept analyses of nurse migration (Freeman et al., 2012) and nurse retention (Efendi et al., 2019) both identified motivation and individual decision as an attribute, signifying that motivation may be an important starting point when exploring retention among nurses who have already migrated (migrant nurses). Attributes are defining characteristics that clarify the meaning of a concept (Walker & Avant, 2019). Motivation is the force underlying overall behavior, action, and intention (Deci & Ryan, 2000; Ratelle et al., 2007). There may be adequate literature describing nurses’ motivations to migrate from source to host countries. However, there is limited discussion on their motivations to remain after arrival in the host countries or whether the motivations that led them to migrate initially will drive them to relocate again to another host country. The onus is to identify the drivers of motivation through a perspective that allows the consideration of migrant nurses’ cultural backgrounds, individual circumstances, and experiences.

Although there are many theories to explain motivation, the SDT may provide a relevant framework for integrating concepts of migration and retention to understand migrant nurses’ motivations. SDT is a broad framework of human motivation proposing that individuals have innate tendencies for learning and well-being, which can either be supported or hindered by social, cultural, or contextual factors (Ryan & Deci, 2000). The four inherent concepts of SDT are motivation, autonomy, competence, and relatedness. Autonomy is volition and the need to own or endorse one’s actions, whereas competence is mastery and the need to succeed and thrive in one’s environment (Deci & Ryan, 2000; Rigby & Ryan, 2018; Ryan & Deci, 2020). Relatedness refers to belongingness and the need to connect with others (Rigby & Ryan, 2018). The Basic Psychological Needs (BPNs) Theory, one component of the SDT, identifies the need for autonomy, competence, and relatedness in health and well-being, where needs satisfaction results in optimal behavior, growth, and high-quality motivation and needs frustration results in vulnerabilities, defensiveness, and overall adverse outcomes (Deci & Ryan, 2000; Legault, 2017; Vansteenkiste & Ryan, 2013).

SDT suggests that the quality of motivation, dependent on whether the needs for autonomy, competence, and relatedness are met, explains why individuals engage in or terminate certain behaviors (Wilson et al., 2008). SDT classifies motivation into autonomous and controlled motivation (Gagné & Deci, 2005). Autonomous motivation involves engagement in activity stemming from one’s sense of choice and full endorsement of actions, whereas controlled motivation involves engagement in action or behavior due to internal or external pressures (Gagné & Deci, 2005; Rigby & Ryan, 2018; Slemp et al., 2020). Central to understanding autonomous and controlled motivation is the varying degrees of regulation in which individuals attempt to assimilate, internalize, or transform behavior into personally endorsed values or actions, subsequently allowing these to originate from their sense of self, resulting in autonomous motivation (Deci & Ryan, 2000; Ryan & Deci, 2000).

SDT conceptualizes a motivation continuum that begins with the lowest form, a complete lack of motivation (amotivation), followed by motivation regulated by various forms of external or internal rewards (extrinsic motivation), leading up to motivation caused by an inherent interest in the activity (intrinsic motivation; Gagné & Deci, 2005; Ryan & Deci, 2020). Autonomous motivation is engagement in an activity either from inherent enjoyment from the activity (intrinsic motivation), from integrating the activity with other aspects of oneself (integrated regulation), or from accepting the underlying value of the activity (identified regulation; Deci & Ryan, 2000; Fernet et al., 2021). Controlled motivation is engagement in an activity because of internal pressures, such as avoidance of negative feelings or achieving a sense of self-worth (introjected regulation) or because of external pressures, such as avoiding punishment or achieving reward (external regulation; Fernet et al., 2021; Gagné & Deci, 2005).

Autonomous motivation often results in better outcomes, including decreased turnover intention, increased commitment, and improved task performance (Austin et al., 2020; Manganelli et al., 2018; Van den Broeck et al., 2021). The strength of the SDT is in providing insight into fostering high-quality motivation to achieve positive outcomes through meeting the BPNs for autonomy, competence, and relatedness (Manganelli et al., 2018).

In the context of SDT, migrant nurses may be motivated to migrate due to financial rewards (external regulation) or to increase self-worth (introjected regulation; Gagné & Deci, 2005); however, after arrival in the host country, when migrant nurses’ needs for autonomy, competence, or relatedness are not satisfied or when their motivations remain controlled (external or introjected regulation), this may eventually lead them to seek employment elsewhere, return to their source country, or proceed in search of another host country. Given the empirical research supporting the SDT, it is natural to assume that high-level motivation may be essential to promote retention among migrant nurses. A study in Canada examining fatigue in newly registered nurses found that somatic factors influenced the quality of motivation. The more the nurses felt fatigued, the more they worked with controlled forms of motivation, increasing their likelihood of burnout (Austin et al., 2020). Another study among nurses in Canada found that autonomous motivation predicted turnover intention and occupational commitment, where nurses working in autonomy-supportive environments show more satisfaction in their work and are less inclined to leave (Fernet et al., 2021).

Organizations that promote work practices and create environments that meet migrant nurses’ needs for autonomy, competence, and relatedness may support migrant nurses in developing high-quality motivation (autonomous motivation) to remain. A systematic review investigating retention among Asian migrant nurses identified career prospects, occupational mobility, career advancement, acculturation, and the availability of support systems as key factors influencing retention (Ung et al., 2024). These factors align with the key concepts of the SDT. Further studies using SDT to explore retention among migrant nurses may support the need for organizations to implement strategies ensuring migrant nurses act under their own free will (autonomy), can practice within their clinical experience (competence), and have good relationships with co-workers (relatedness).

## Nurse Migration, Retention, and Culture

Another important consideration in studying migrant nurses and their motivations is their cultural background. It is unclear whether the theories mentioned earlier are relevant to nurses from different cultural backgrounds. Hofstede (1980) postulated that the differences in motivations, management styles, and organizational structures might be attributed to cultural differences; thereby, organizational theories, most of which were developed from the Western perspective, may only apply to some cultures. Hofstede’s question, “Do American theories apply abroad?” (Hofstede, 1980, p. 42) led him to establish the continuum of collectivism and individualism as one of the many ways to simplify the complex construct of culture (Jackson, 2020; Minkov et al., 2017). Culture is a shared pattern of beliefs, norms, and values of people in a society (Hofstede, 1980; Triandis, 1995). This may mean that policies developed from organizational theories of a specific culture may be ineffective in another cultural environment (Hofstede, 1980). For instance, Fish et al. (2021) explored burnout among nurses from China and Australia and identified that nurses from Australia were more at risk for burnout than nurses from China. This was attributed to collectivist values wherein nurses from China were more accepting of formal leadership and valued respect for their superiors, which played a protective role against burnout and increased workplace resilience (Fish et al., 2021). A scoping review on workplace bullying in nursing identified differences in perceptions of, and responses to, bullying across various cultural clusters (Karatuna et al., 2020). These differences suggest cultural factors may be crucial when developing or improving organizational practices.

Collectivist cultures place importance on the goals of their group, view themselves as interdependent, and prioritize relationships and conformity. In contrast, individualist cultures place importance on personal goals, view themselves as independent, and prioritize personal freedom (Triandis, 1995). These stark differences have implications for studies involving individuals of diverse cultures, given that what one culture values as necessary may not be as important in another. Research suggests that Maslow’s hierarchy of needs varies across cultures such that collectivist cultures’ basic need is belongingness in comparison to individualist cultures’ basic needs of food, water, and shelter (Gambrel & Cianci, 2003; Nevis, 1983; Papaleontiou-Louca et al., 2022). Individuals can also be motivated by higher-level needs regardless of whether their lower-level needs are satisfied (Desmet & Fokkinga, 2020). As such, nurse retention practices aimed at meeting basic physiological needs from Maslow’s perspective may not apply to nurses from collectivist cultures.

Like Maslow’s hierarchy of needs, Herzberg’s motivation and social exchange may not be applicable when considering cultural factors. Motivators in one culture may be considered demotivators in another, or they may not have the expected effect on job satisfaction or motivation for all employees (Alrawahi et al., 2020; Matei & Abrudan, 2016; Sledge et al., 2008). Furthermore, the emphasis on the hierarchy and the importance of relationships and social exchange may differ across cultural settings (Chernyak-Hai & Rabenu, 2018; Deng et al., 2021). The differences across collectivist and individualist cultures of what may be seen as motivators or hygiene factors or positive or negative social exchange imply that these theories may be ineffective or at least partially effective in supporting retention strategies among migrant nurses.

The inclusion of cultural background in studies of retention among migrant nurses is another strength of the SDT. According to the SDT, BPNs are universal, and the needs for autonomy, competence, and relatedness to foster positive outcomes are significant regardless of culture (Deci & Ryan, 2000; Magson et al., 2022). The importance of BPNs in influencing work engagement, job satisfaction, and turnover intention has been studied on nurses from various cultures, including research conducted in Eastern (Hwang et al., 2022; Ni et al., 2023), Western (Boudrias et al., 2020; Klein, 2017), and African countries (Liebenberg et al., 2022; Onyishi et al., 2019). The satisfaction of BPNs increased work engagement among nurses in China (Ni et al., 2023), career satisfaction among nurses in Nigeria (Onyishi et al., 2019), and decreased turnover intention among nurses in the United States (Klein, 2017). In a study conducted in Korea, Hwang et al. (2022) found that nurses had an increased turnover intention when BPNs were unmet. Meanwhile, Liebenberg et al. (2022) found that among the three BPNs, only competence influenced work engagement among nurses in South Africa. Liebenberg et al. (2022) elaborate that the complex issue of professional autonomy in nursing and the differences in perception of social support across cultures may explain these findings. Similarly, satisfaction with the need for autonomy and competence, but not relatedness, was found to facilitate nurses’ adaptation to their work environment in Canada (Boudrias et al., 2020) and decrease compassion fatigue among nurses in the United States (Klein, 2017). These studies demonstrate that while BPNs were consistent across cultures, variations still exist, possibly due to the contextual differences in the study settings.

Examining culture using the dichotomy of individualism and collectivism may not always be appropriate due to possible heterogeneity within cultures (Fatehi et al., 2020). Although some scholars propose extending the dichotomy to multiple dimensions, others believe in the coexistence of values of individualism and collectivism across cultures and individuals (Fatehi et al., 2020; Tamis-LeMonda et al., 2008). The coexistence is believed to be dynamic and may change depending on social, political, situational, and economic contexts (Tamis-LeMonda et al., 2008). The dichotomy of individualism and collectivism places autonomy and relatedness at opposite ends of the spectrum (Tamis-LeMonda et al., 2008). Although the dichotomy and fluidity of the concepts present limitations, the inclusion of both autonomy and relatedness as BPNs captures the importance of both values, thus maintaining the SDT’s relevance in studies involving multiple cultures. However, it is essential to note that SDT defines autonomy as self-endorsement of one’s actions rather than independence (Deci et al., 2001).

Research has supported the cross-cultural universality of the needs for autonomy, competence, and relatedness in other contexts. These include cross-cultural research establishing that satisfaction of needs for autonomy, competence, and relatedness contributes to well-being and happiness (Chen et al., 2015; Martela et al., 2022), promotes student achievement and life satisfaction (Nalipay et al., 2020; Sheldon et al., 2009), promotes motivational outcomes in teachers (Kaplan & Madjar, 2017), and predicts behavior and motivation in organizations (Deci et al., 2001; Magson et al., 2022) and in sport (Quested et al., 2013). The robust evidence supporting SDT and the universality of the needs for autonomy, competence, and relatedness in promoting high-quality motivation and good outcomes across cultures supports the applicability of SDT in studying retention among migrant nurses.

## Conceptual Framework to Study Retention Among Migrant Nurses

**Figure 1.**
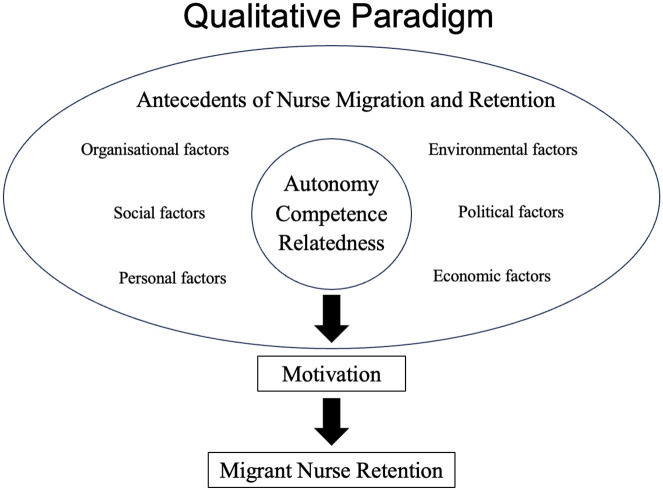
Conceptual Framework: The Relationship of Nurse Migration, Retention, and SDT, Situated in a Qualitative Paradigm *Note.* The figure displays antecedents of nurse migration and retention and how they subsequently influence migrant nurses’ needs for autonomy, competence, and relatedness. Satisfaction of autonomy, competence, and relatedness leads to high-quality motivation, ultimately contributing to migrant nurse retention. The relationships are situated within a qualitative paradigm to allow full exploration of the context of experiences and perceptions of migrant nurses. Adapted from Deci and Ryan (2000), Efendi et al. (2019), and Freeman et al. (2012).

Understanding the intersections of BPNs, migration, and retention is essential to foster high-quality motivation among migrant nurses. To enable researchers to fully explore how migration experiences and antecedents satisfy migrant nurses’ BPNs and how these influence their motivations to stay, this paper suggests situating the SDT framework within a qualitative paradigm, specifically a constructivist paradigm (Figure 1).

Antecedents refer to events or incidents that precede the concept’s occurrence (Fitzpatrick & McCarthy, 2016; Walker & Avant, 2019). Antecedents of nurse migration include social, personal, political, economic, and educational pull factors from host countries and push factors from source countries that motivate nurses to migrate (Freeman et al., 2012). Meanwhile, the antecedents of nurse retention are organizational, social, personal, economic, and environmental factors that motivate nurses to remain (Efendi et al., 2019). Considering how antecedents meet the BPNs for autonomy, competence, and relatedness may contribute to a more nuanced understanding of motivation for retention among migrant nurses.

The constructivist paradigm recognizes that realities are individually created mental constructions and that individuals interpret and make meaning of their experiences within a particular context (Denicolo et al., 2016; Guba & Lincoln, 1994). This framework allows researchers to understand the meanings of the context (BPNs for autonomy, competence, and relatedness) as constructed by migrant nurses and how their experiences and these meanings shape their perceptions, actions, and motivations (intentions to stay). This allows researchers to comprehend how migrant nurses perceive their BPNs and how these influence their motivations to stay.

A number of studies on migrant nurse retention and turnover have used quantitative methodologies using validated or researcher-developed questionnaires to measure outcomes (Villamin et al., 2024). However, quantitative methods do not allow the full exploration of participants’ experiences and so do not discover reasons for patterns of behavior (Busetto et al., 2020). Migration is a complex phenomenon, where migrant nurses often experience stressful events before, during, and after their move, affecting their psychological well-being and their personal and professional experiences (Bhugra & Becker, 2005; Ng Chok et al., 2018; Torres Fernández et al., 2017). Qualitative methods will allow researchers to generate data on events or experiences from the migrant nurses’ perspectives, staying close to the data and gaining a comprehensive understanding of migration experiences that lead to retention (Doyle et al., 2020; Sandelowski, 2000).

There is a multitude of studies describing the experiences of transition, migration, acculturation, and workforce integration of migrant nurses (Chun Tie et al., 2018; Montayre et al., 2018; Moyce et al., 2016; Ng Chok et al., 2018; Pressley et al., 2022; Safari et al., 2022), with only one study reporting specifically on retention (Pressley et al., 2022). Nonetheless, the detailed descriptions in these studies support the merit of viewing migrant nurse retention through a qualitative paradigm.

## Recommendations for Practice and Future Research

This paper provides another perspective on exploring nurse migration and retention. It suggests that the SDT framework can provide a comprehensive understanding of motivations regardless of cultural background, which may be vital in migration and retention studies. Although this paper focused on migrant nurses, the robust evidence behind the SDT suggests that it can be applied in studies involving broader migrant workforce retention.

The SDT framework also explains how the work environment supports employee motivation and well-being (Deci et al., 2017), which is linked to job satisfaction and retention. In practice, identifying how migrant nurses (or other migrant employees) perceive their needs for autonomy, competence, and relatedness to be satisfied may assist organizations and policymakers in making informed decisions that promote autonomous motivation, which is linked to the overall employees’ well-being and retention. Migrant nurses (or migrant employees in general) may face unique circumstances that challenge their needs for autonomy, competence, and relatedness. Thus, organizations may need to modify practice environments or create conditions that promote meeting their needs rather than taking one-size-fits-all retention strategies.

Retention strategies for migrant nurses, in line with the SDT perspective, aim to meet migrant nurses’ needs for autonomy, competence, and relatedness. Previous studies identified migrant nurses’ challenges with communication, skill transfer, differences in professional practice and role expectations, cultural adjustment, and social isolation, which often hinder transition, acculturation, and retention (Pressley et al., 2022; Randall & De Gagne, 2023; Safari et al., 2022). Hence, organizations should consider reviewing orientation programs for migrant nurses to ensure these are tailored to address their needs while promoting conditions that encourage clinical decision-making to satisfy their need for autonomy. In addition, organizations should support access to ongoing training and mentorship programs, pathways to transfer and practice clinical skills, and opportunities for professional growth to satisfy migrant nurses’ need for competence. Supportive working environments that include formal mentorship and peer support within and outside the workplace may satisfy their need for relatedness. As there are limited studies applying SDT to migrant nurses, further research incorporating SDT and retention may uncover new areas not previously investigated, such as autonomy-supportive environments as defined by migrant nurses and how modifying these environments and implementing relevant strategies lead to retention and positive migration outcomes.

## Conclusion

A persistent deficit in the nursing workforce threatens global health, and the increasing trend of nurse migration further compounds the intricacies of this shortfall. Understanding the experiences of migrant nurses and the experiences that motivate them to remain in the host country is essential in addressing global nursing workforce challenges and promoting retention. Migration and retention theories are valuable in their disciplines; however, these theories have limitations as they are not used in the presence of another; that is, retention theories are not often used as a framework to study migrant nurses and migration theories are seldom used to research retention. In addition, the most commonly used theories may not always apply to nurses from different cultural backgrounds. An approach that includes migration and retention concepts is required to understand retention among migrant nurses.

Viewing the retention of migrant nurses from the SDT’s perspective within a qualitative paradigm allows the exploration of concepts of migration and retention from the perspectives of migrant nurses. SDT provides a comprehensive understanding of human motivation supported by robust empirical research linking its significance to motivation and overall positive outcomes while being applicable in both collectivist and individualist cultures. The proposed conceptual framework, inclusive of the SDT, provides a foundation for the exploration of how antecedents of both migration and retention satisfy migrant nurses’ BPNs for autonomy, competence, and relatedness and how this results in motivations that ultimately lead to retention.
